# Emphysematous pyelonephritis-related delayed abdominal wall necrotizing fasciitis: A case report

**DOI:** 10.1097/MD.0000000000050601

**Published:** 2026-09-04

**Authors:** Jacqueline Yu-Min Beh, Tung-Mao Lai, Dun-Hao Chang

**Affiliations:** a Department of Plastic and Reconstructive Surgery, Far Eastern Memorial Hospital, New Taipei, Taiwan; b School of Medicine, National Yang Ming Chiao Tung University, Taipei, Taiwan; c Department of Information Management, Yuan Ze University, Taoyuan, Taiwan.

**Keywords:** case reports, debridement, emphysematous pyelonephritis, necrotizing fasciitis, retroperitoneal space

## Abstract

**Rationale:**

Emphysematous pyelonephritis (EPN) is a severe, gas-forming renal infection typically associated with diabetes mellitus. Necrotizing fasciitis (NF) is a rapidly progressive soft tissue infection with significant morbidity. Although concurrent EPN and NF have been described, delayed NF arising years after previously treated EPN is exceedingly rare. This report highlights a unique case of abdominal wall NF secondary to a chronic infectious pathway originating from prior EPN.

**Patient concerns:**

A 55-year-old woman with poorly controlled type II diabetes mellitus initially presented with right flank pain, fever, and malaise due to EPN. Two years later, she developed progressive swelling, pain, and discoloration over the right lower abdominal wall, hip, and gluteal region.

**Diagnoses:**

Imaging at initial presentation confirmed right-sided EPN with hydronephrosis requiring percutaneous drainage. At the delayed presentation, computed tomography revealed gas-forming inflammation extending from the right kidney through the retroperitoneum to the abdominal wall and gluteal region. A retained double-J stent and a suspected fistulous communication suggested a chronic infectious source leading to NF.

**Interventions:**

The patient underwent emergent surgical debridement, followed by serial debridements (eight sessions), broad-spectrum antibiotics, negative-pressure wound therapy, and hyperbaric oxygen therapy. After achieving healthy granulation tissue, wound closure was accomplished using an adhesive-based shoelace device.

**Outcomes:**

The infection gradually resolved, and the wound healed completely after staged closure. The patient recovered fully and was discharged after 58 days of hospitalization.

**Lessons:**

Clinicians should remain alert to the possibility of chronic or delayed soft tissue infection in patients with prior EPN, particularly when indwelling urinary stents remain in place. Long-term surveillance is crucial to confirm definitive source control and to prevent catastrophic complications such as NF.

## 1. Introduction

Emphysematous pyelonephritis (EPN) is a life-threatening necrotizing infection of the renal parenchyma characterized by gas formation within the collecting system or perinephric tissues. It occurs most frequently in patients with uncontrolled diabetes mellitus and carries a high risk of sepsis and mortality.^[1]^ Necrotizing fasciitis (NF) is another rapidly progressive and destructive soft tissue infection that typically involves the fascia and subcutaneous layers. Although EPN and NF share overlapping risk factors, including immunocompromised status and hyperglycemia, their occurrence as sequential pathologies is exceedingly uncommon.

Most reported cases describe simultaneous or early-onset NF associated with EPN, believed to result from direct extension of infection through retroperitoneal fascial planes.^[2,3]^ However, delayed NF developing years after apparent resolution of EPN has not been well documented. We present an unusual case of abdominal wall and gluteal NF emerging 2 years after treatment for EPN, likely due to a chronic infectious tract associated with a retained ureteral stent. This case underscores the importance of long-term surveillance following EPN in high-risk patients.

## 2. Case presentation

A 55-year-old woman with a long-standing history of poorly controlled type II diabetes mellitus presented with a 1-week history of right flank pain and malaise. She had been treated empirically with oral antibiotics at a local clinic before arrival. On admission, she was afebrile but demonstrated right costovertebral angle tenderness. Laboratory tests revealed leukocytosis (27,810/μL), hyperglycemia (602 mg/dL), elevated creatinine (1.57 mg/dL), and elevated C-reactive protein (22.67 mg/dL). Urinalysis showed pyuria and bacteriuria.

**Figure 1.**
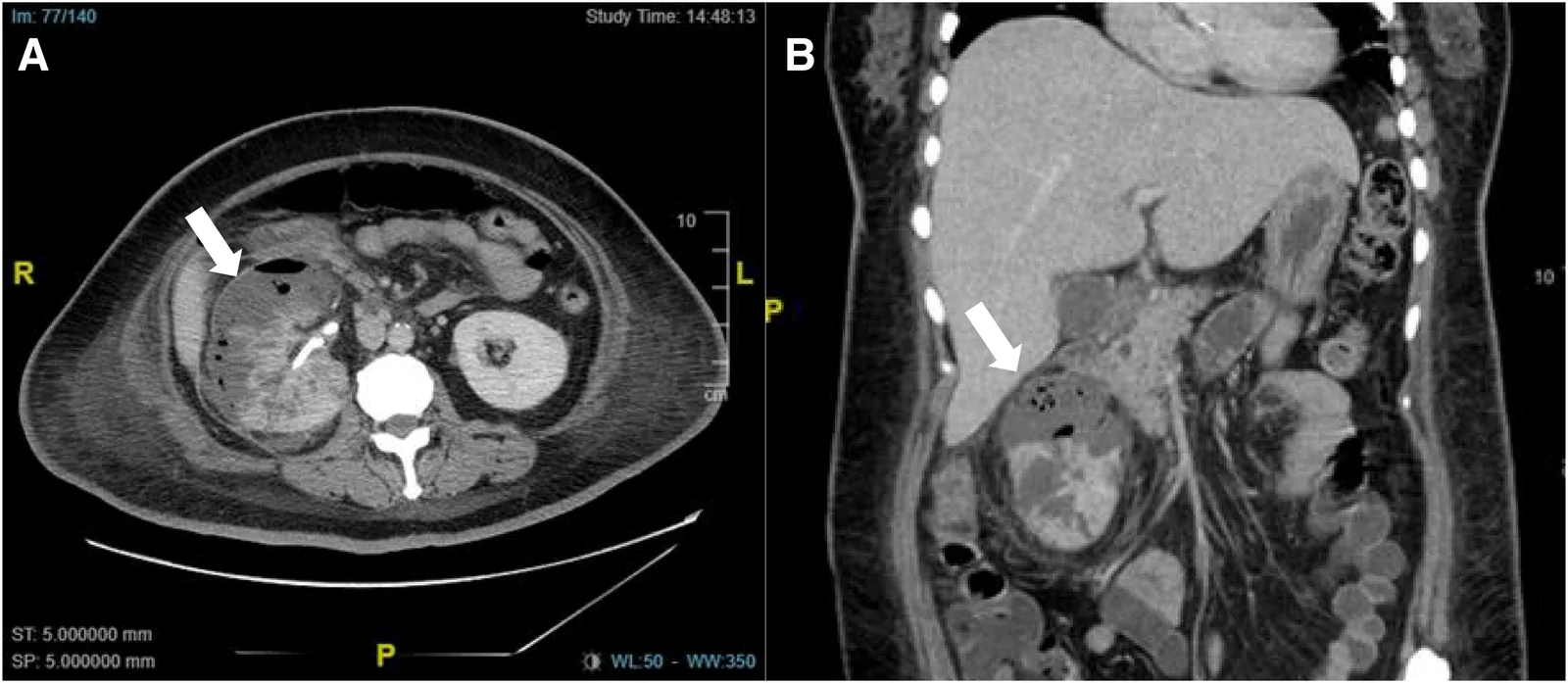
Computed tomography of the right emphysematous pyelonephritis. (A) Axial view showing gas formation within the right renal parenchyma and perinephric space (arrow). (B) Coronal view confirming emphysematous changes in the right kidney with perinephric fluid collection.

Computed tomography (CT) of the abdomen demonstrated right-sided acute pyelonephritis with mild hydronephrosis secondary to obstruction at the ureteropelvic junction. She underwent percutaneous nephrostomy (PCN) for abscess drainage and short-term double-J catheter insertion (6Fr, 26cm, Bard inlay optima stent) for temporary urinary drainage. Cultures of drained abscess grew *Enterobacter aerogenes*. After 4 days of broad-spectrum antibiotics (Ceftriaxone 1000 mg Q12H), the nephrostomy tube yielded minimal drainage. The follow-up CT revealed emphysematous abscess formation extending to the perinephric space, indicating PCN malfunction (Fig. 1). Therefore, the PCN tube was removed, and 2 additional pigtail drainage catheters were inserted. After 2 weeks of drainage and antibiotic treatment, the infection subsided, and the drains were removed. She was then discharged with oral antibiotics. A urology clinic visit was scheduled to arrange the double J stent removal; however, she was lost to follow-up thereafter.

**Figure 2.**
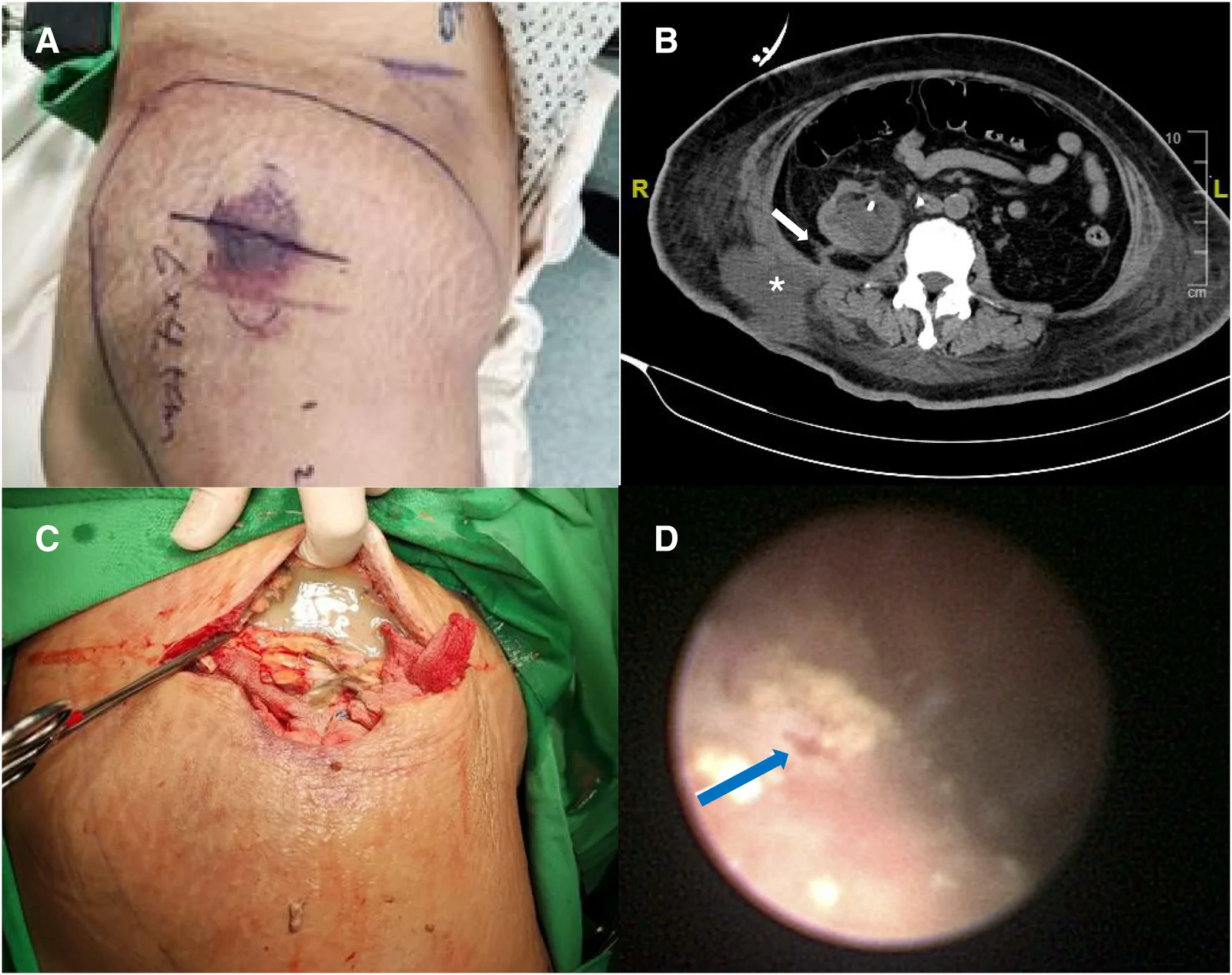
Findings 2 years after the prior emphysematous pyelonephritis episode. (A) Clinical photograph showing right hip induration, skin discoloration, tenderness, and crepitus. (B) Non-contrast CT scan demonstrating a posterolateral abdominal wall abscess (asterisk) with a suspected tract connecting to the right kidney (arrow), consistent with a delayed infectious extension. (C) Intraoperative findings revealing extensive pus accumulation and free air within the muscular fascia during surgical exploration. (D) Cystoureteroscopy revealing yellowish-white encrustation with stone formation at the right side ureter and a mucosal defect (arrow) which might be a fistula. CT = computed tomography.

Two years later, she presented with progressive swelling, pain, and discoloration of the right gluteal region and lower abdominal wall (Fig. 2A). Physical examination revealed induration, erythema, and tenderness extending to the hip and upper thigh. Laboratory values showed leukocytosis (22,030/μL), elevated creatinine (4.29 mg/dL), and markedly elevated C-reactive protein (30.36 mg/dL). Non-contrast CT demonstrated extensive gas-forming inflammation tracking from the right kidney through the retroperitoneum into the abdominal wall and gluteal region. A suspected fistulous communication between the perinephric region and the abdominal wall abscess was identified, suggesting a chronic infectious pathway originating from prior EPN (Fig. 2B).

Broad-spectrum antibiotics (Ceftaroline 400 mg Q8H and Metronidazole 500 mg Q8H) were initiated. Emergent surgical debridement revealed extensive necrosis of the fascia and intermuscular tissue, with purulent tracking along deep planes (Fig. 2C) Cystoureteroscopy revealed a retained double-J ureteral stent with marked yellowish-white encrustation and stone formation and a mucosal defect which might be a fistula (Fig. 2D). Fibrosis and stricture of the right upper ureter and ureteropelvic junction were observed, accompanied by turbid urine and hematoma accumulation. Endoscopic lithotripsy and removal of the retained double-J stent were successfully performed, but no ureteral stent or nephrostomy tube was placed because of the ongoing severe infection, despite mild hydronephrosis being noted. She underwent temporary hemodialysis for acute kidney injury and oliguria.

Pus cultures grew Enterococcus faecalis, Streptococcus constellatus, and Escherichia coli. The patient underwent 8 more sessions of debridement, and negative-pressure wound therapy (NPWT) was applied continuously at −125 mm Hg for 2 weeks, with dressing changes every 3 to 4 days. Hyperbaric oxygen therapy was administered at 2.5 ATA for 120 minutes per session, once daily, for a total of 10 sessions.

**Figure 3.**
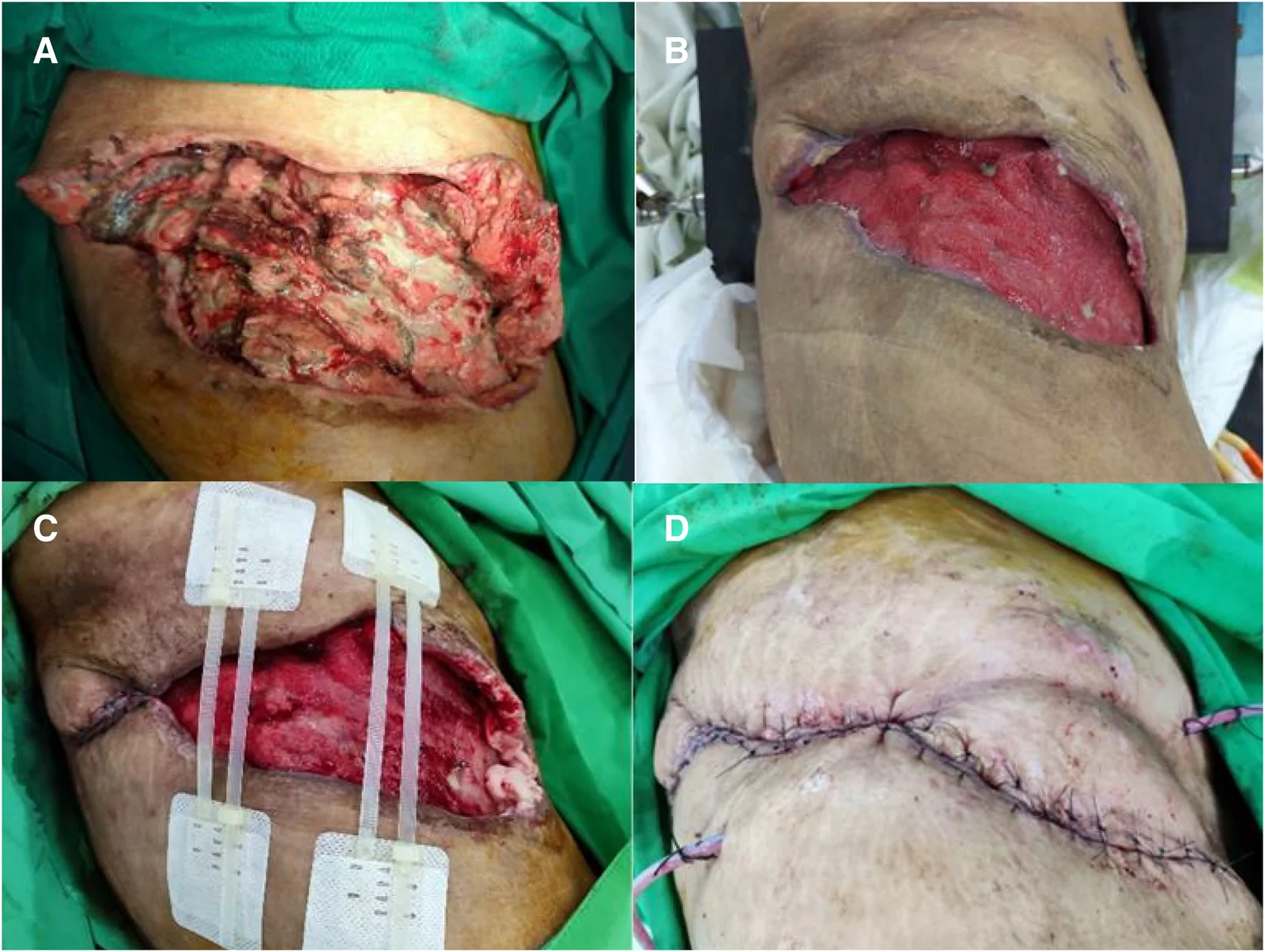
Subsequent wound management of the abdominal wall necrotizing fasciitis. (A) Necrotic wound bed after the second debridement. (B) Well-granulated wound bed following negative-pressure wound therapy. (C) Wound approximation using the adhesive-based closure system (EZip®). (D) Primary closure after 2 weeks of wound advancement.

After healthy granulation tissue developed, wound approximation was achieved using an adhesive-based shoelace closure system (EZip®), followed by primary closure (Fig. 3). The creatinine level decreased to 1.78mg/dL, with recorded urine output >1cc/kg/hour, and she was discharged after 58 days of hospitalization. No stent or Foley catheter was retained upon discharge. The wound healed well at 1 month follow-up at the clinic, and she reported normal voiding without urinary symptoms. Then she was lost to follow-up again and didn’t return to the clinic anymore. This case report was approved by the Research Ethics Review Committee of Far Eastern Memorial Hospital (protocol code 114112-C, date of approval: June 16, 2025). The requirement for written informed consent was waived by the Research Ethics Review Committee because the report involved retrospective review of existing clinical data and contained no identifiable patient information.

## 3. Discussion

EPN is a life-threatening necrotizing infection of the renal parenchyma and surrounding tissues that predominantly affects patients with poorly controlled diabetes mellitus. Gas production by facultative anaerobic organisms, impaired tissue perfusion, urinary tract obstruction, and compromised host immunity all contribute to its pathogenesis and account for its substantial morbidity and mortality. Early diagnosis with CT, prompt urinary drainage, appropriate antimicrobial therapy, and adequate source control are the cornerstones of successful management.

The radiological severity of EPN is commonly classified according to the CT-based system proposed by Huang and Tseng, which categorizes disease into Class 1 (gas confined to the collecting system), Class 2 (gas confined to the renal parenchyma), Class 3A (extension of gas or abscess to the perinephric space), Class 3B (extension to the pararenal space), and Class 4 (bilateral EPN or EPN in a solitary kidney).^[4]^ In our patient, the initial episode was most consistent with Class 3A EPN as gas-containing infection and abscess formation extended beyond the renal parenchyma into the perinephric space. Interestingly, despite apparent clinical resolution after drainage and antibiotic therapy, the patient later developed delayed retroperitoneal infection with abdominal wall NF 2 years later. This progression represents a secondary delayed complication rather than progression of the original EPN classification. This case illustrates that even when the acute infection appears clinically controlled, inadequate long-term follow-up may leave persistent risk factors unrecognized.

NF secondary to EPN is uncommon and is generally reported during the acute phase of infection through direct extension along retroperitoneal fascial planes or anatomical weak points, such as the inferior lumbar triangle.^[5–8]^ In contrast, delayed NF developing 2 years after the initial episode of EPN has rarely been reported. In the present case, CT demonstrated inflammatory extension from the right kidney to the abdominal wall, and cystoureteroscopy identified a retained double-J ureteral stent with marked encrustation and ureteral stricture. Although a persistent fistulous communication could not be definitively confirmed because dedicated fistulography or retrograde pyelography was not performed, these findings suggest the presence of a chronic infectious pathway linking the urinary tract to the retroperitoneal soft tissues.

The retained ureteral stent likely played an important role in this patient’s delayed presentation. Forgotten ureteral stents are well recognized to increase the risks of bacterial colonization, encrustation, obstruction, recurrent urinary tract infection, and deterioration of renal function.^[9]^ Although a direct causal relationship between the retained stent and the subsequent NF cannot be conclusively established in this case, prolonged indwelling of the stent may have promoted chronic inflammation and persistent infection, ultimately facilitating retroperitoneal extension into the abdominal wall. This case therefore highlights the importance of structured surveillance systems and timely removal of temporary ureteral stents after EPN treatment.

Successful management required a multidisciplinary approach. Prompt and repeated surgical debridement remains the cornerstone of NF treatment, while broad-spectrum antimicrobial therapy provides systemic infection control. Adjunctive negative-pressure wound therapy promoted wound-bed preparation and exudate control, whereas hyperbaric oxygen therapy may have further enhanced bacterial clearance, tissue oxygenation, and granulation tissue formation. Definitive closure was performed only after complete control of local infection and the establishment of a healthy granulating wound bed, resulting in satisfactory wound healing without early recurrence.

This case report has several limitations. First, differential renal function was not assessed; therefore, the functional status of the affected kidney could not be fully evaluated. Second, although the patient showed satisfactory wound healing and renal recovery at the 1-month follow-up, she was subsequently lost to follow-up. Consequently, long-term outcomes, including definitive resolution of the infection, recurrence status, renal function, and persistence of any residual fistulous communication, could not be confirmed.

## 4. Conclusion

In conclusion, this case emphasizes that delayed NF should remain a diagnostic consideration in patients with a history of EPN who present with new soft tissue infection, particularly when temporary urinary drainage devices have not been appropriately removed. Careful long-term follow-up, confirmation of definitive source control, and timely management of retained urinary devices are essential to prevent potentially life-threatening delayed infectious complications.

## Author contributions

**Data curation:** Jacqueline Yu-Min Beh, Dun-Hao Chang.

**Formal analysis:** Jacqueline Yu-Min Beh, Tung-Mao Lai, Dun-Hao Chang.

**Validation:** Jacqueline Yu-Min Beh.

**Conceptualization:** Tung-Mao Lai, Dun-Hao Chang.

**Investigation:** Tung-Mao Lai, Dun-Hao Chang.

**Writing – review & editing:** Tung-Mao Lai, Dun-Hao Chang.

**Writing – original draft:** Jacqueline Yu-Min Beh.
